# Conjunctival mast cell as a mediator of eosinophilic response in ocular allergy

**Published:** 2008-08-22

**Authors:** Dai Miyazaki, Takeshi Tominaga, Keiko Yakura, Chuan-Hui Kuo, Naoki Komatsu, Yoshitsugu Inoue, Santa J. Ono

**Affiliations:** 1 Division of Ophthalmology and Visual Science, Faculty of Medicine, Tottori University, Yonago, Japan; 2 Emory University School of Medicine and Emory Eye Center, Dobbs Ocular Immunology Laboratories, Atlanta, Georgia

## Abstract

**Purpose:**

To determine the contribution of conjunctival mast cells to the allergen-specific inflammatory responses in eyes with allergic conjunctivitis and to test the hypothesis that mast cells act as mediators of the early phase response.

**Methods:**

The participation of mast cells in allergen-induced inflammatory cell recruitment was studied in an experimental murine model of allergic conjunctivitis. Experimental allergic conjunctivitis was induced by a single or multiple sensitizing injections of an allergen. The conjunctiva of allergen-sensitized, mast cell-deficient (*Kit^w^/Kit^w-v^*) mice were reconstituted with conjunctival mast cells isolated from naïve wild type mice by subconjunctival transfer. *Kit^w^/Kit^w-v^* mice and conjunctival mast cell reconstituted *Kit^w^/Kit^w-v^* mice were evaluated for early phase reactions and late phase inflammatory responses.

**Results:**

The early phase response was minimal in *Kit^w^/Kit^w-v^* mice after both a single injection and multiple sensitization injections of the allergen. The early phase responses were fully restored following adoptive transfer of isolated conjunctival mast cells from naïve wild type mice. Eosinophilic inflammatory responses were significantly depressed in *Kit^w^/Kit^w-v^* mice without the impairment of allergen-specific priming. Reconstitution of the conjunctiva of *Kit^w^/Kit^w-v^* mice with mast cells from wild type mice fully restored the allergen-specific eosinophilic responses but not the neutrophilic responses.

**Conclusions:**

Our data indicate that conjunctival mast cells are essential for eosinophilic inflammation but not for neutrophilia in allergic conjunctivitis that is mediated by mast cell activation.

## Introduction

Allergic diseases affect approximately one-third of the population and constitute one of the major health care problems in the Western world [1]. Ocular allergy is an example of an IgE-mediated immediate hypersensitivity reaction. These reactions are initiated by the cross-linking of IgE by an allergen, and their effects are mediated by the degranulation of mast cells. Immediate hypersensitivity reactions are characterized by early phase and late phase responses. The early phase response develops immediately after the exposure to the allergen, and clinical symptoms and signs such as itching, chemosis, and congestion are manifested very quickly. This is followed by the late phase response after 8-24 h, which is characterized by conjunctival eosinophilia and neutrophilia.

Eosinophilic inflammation is not only a hallmark of allergic conjunctivitis but also a major cause for tissue injury and remodeling. Therefore, to understand how this response develops or is exacerbated is important in developing an effective strategy to combat ocular allergy.

The role played by T cells in the eosinophilic inflammation of severe allergic conjunctivitis has been gaining interest. For example, we have reported that the potent inhibitor of T cell activation, tacrolimus, will reduce the symptoms and signs of a severe form of allergic conjunctivitis involving a corneal ulcer [2]. In experimental settings, an eosinophil infiltration can occur independent of the presence of mast cells [3,4]. Thus, the question arises as to how mast cells contribute to ocular inflammatory cell recruitment. Are conjunctival mast cells simply serving to induce the clinical symptoms during the early phase and play a subordinate part in the final late phase inflammatory process?

To answer this question and to evaluate the contribution of mast cells to the late phase inflammatory process, we have developed a mast cell reconstituted model. Bone marrow-derived mast cells (BMMCs) are commonly used to restore mast cell populations. However, these cells are not appropriate for the analyses of ocular responses because BMMCs have a distinct lineage from mast cells in connective tissues such as those residing in the conjunctiva [5]. Indeed, important maturation markers of connective tissue type mast cells such as CCR3, a receptor of eotaxin-1, are not expressed by BMMCs [5]. For inflammatory responses, mast cells are recruited to the effector site from the bone marrow, and they mature in situ presumably by differentiating into an inflammatory phenotype by chemokine receptors. Thus, conjunctival allergic responses are mixed phenomena induced by tissue-residing mast cells and newly recruited mast cells.

We have developed an adoptive transfer model in which the conjunctiva of mast cell-deficient mice is reconstituted with conjunctival mast cells from wild type mice. After the induction of allergen-specific allergic conjunctivitis, we carefully assessed the contribution of mast cells to the early phase and late phase responses.

## Methods

### Animals

Mast cell-deficient mice were of the *Kit^w^/Kit^w-v^* strain. c-Kit is a protein that is essential for the development and activation of mast cell lineage cells. *Kit^w^/Kit^w-v^* mice carry point mutations in the transmembrane and kinase domains and therefore lack the ability to activate the signaling cascade initiated by its ligand, stem cell factor (SCF). The mast cell deficiency is greater than 99% in the systemic circulation, airway, intestine, and skin of these mice [6]. The mast cell-deficient WBB6F_1_-*Kit^w^/Kit^w-v^* (*Kit^w^/Kit^w-v^*) mice and congenic wild-type (WBB6F_1_^+/+^) mice were purchased from Shimizu Laboratories Supplies (Kyoto, Japan). The wild type mice were age-matched and gender-matched and reared under identical conditions as the mast cell-deficient *Kit^w^/Kit^w-v^* mice. The procedures used conformed to all of the regulations for laboratory animal research outlined by the Animal Welfare Act, the Department of Health, Education, and Welfare (NIH) guidelines, and the ARVO statement for the experimental use of animals.

### Immunization and induction of experimental allergic conjunctivitis

*Kit^w^/Kit^w-v^* and wild type mice were allergen-sensitized using protocols developed in our laboratory [7-9]. We used two established immunization protocols; one used a single exposure and the other using multiple exposures [7-9]. In both models, the inflammatory cell recruitment in the late phase is dependent on mast cell degranulation.

For the single exposure protocol [8], anesthetized mice were injected with a suspension of 50 μg of ragweed pollen (ICN, Aurora, OH) and 1 mg of aluminum hydroxide (Sigma, St. Louis, MO) into the left hind footpad. On day 22, conjunctivitis was induced by topical application of 1.5 mg of ragweed suspended in 10 μl of phosphate buffered saline (PBS). Control mice were mock-sensitized with aluminum hydroxide and challenged identically with the ragweed suspension.

The clinical responses were graded using a modified version of our published method 20 min after the allergen challenge [7-9]. Conjunctival edema, lid edema, tear/discharge, and conjunctival redness were graded from 0 to 4 by an observer who was masked to the treatment protocol of the mice (Table 1). The cumulative clinical score was calculated as the sum of the scores of each of the four parameters with a range from 0 to 16.

**Table 1:** Grading criteria of clinical scores.

**Criteria **	**0**	**1**	**2**	**3**	**4**
conjunctival edema	none	focal edema	edema confined within one quadrant	edema extending to 3 quadrants	massive edema.
lid edema	none	slightly narrowed palpebral fissure (3/4 normal width) with congestionsevere edema (1/3 of normal width)	narrowed palpebral fissure with edema (2/3 of normal width)	narrowed fissure with severe edema (1/3 of normal width)	massive edema (cornea barely visible).
tear/discharge	minimal level of tear meniscus	increased tear level with concave meniscus	increased tear level with convex meniscus	highly increased tear level with mucous secretion	excessive tearing with copious discharge
conjunctival redness	none	barely detectable venous dilatation	three or four dilated vessels at corneal limbus	five or six dilated vessels at limbus	marked ciliary injection

The clinical responses were graded at 20 min after allergen challenge.

For the repeated exposure protocol, groups of mice (10 mice per group) were initially injected intraperitoneally with 1 mg of aluminum hydroxide that was conjugated with cat dander extract (200 BAU/mouse; ALK Laboratories, Horsholm, Denmark) on days 1, 14, and 24. Control mice were injected with the vehicle of the dander extract on the same days. Concomitantly, aluminum hydroxide (25 μg/eye)-conjugated cat dander extract (10,000 BAU/ml) or vehicle was applied topically to the eye on days 1, 2, 3, 7, and 14. Thereafter, mice were exposed to the allergen for sensitization once a week by topical instillation of cat dander extract (10,000 BAU/ml) or vehicle onto the eye. Eight weeks after the initial sensitization, affinity-purified Fel d 1 (0.5 mg/ml), cat dander extract (10,000 BAU/ml; Greer Laboratories, Lenoir, NC), or vehicle was applied to the eyes (10 μl /eye) for two consecutive days for the final priming. The early phase and late phase responses were elicited by instilling cat hair allergen (Fel d 1) to all mice 24 h after the last allergen sensitization.

For histological evaluation, mice were sacrificed 2 h after the challenge to investigate the early phase response and 24 h after the challenge to examine the late phase inflammatory response. The tissues from the eyes were fixed in 4% paraformaldehyde and embedded in HistoResin (Leica Instruments GmbH, Heidelberg, Germany). Serial sagittal sections (3 μm thick) were cut and stained with toluidine blue, Giemsa, or hematoxylin and eosin. Three serial sections of the conjunctival tissue were examined from each eye to determine the number of inflammatory cells recruited into the conjunctiva. A masked observer counted the number of cells under a 400X microscopic field.

### Measurement of allergen-specific IgE and IgG1 antibodies

Sera were collected by cardiac puncture 24 h after the allergen challenge. The Fel d 1-specific IgE and IgG1 levels were measured by ELISA [9,10]. The sera were pipetted onto a Fel d 1-coated microtiter plate (Maxisorp; Nalge Nunc International KK, Tokyo, Japan), incubated with biotin-conjugated anti-IgE (BD Biosciences, Franklin Lakes, NJ) or anti-IgG_1_ (SouthernBiotech, Birmingham, AL) antibodies, and developed for peroxidase-based substrate detection.

### Isolation and FACS analysis of mast cells from the conjunctiva

The protocol to isolate crude conjunctival mast cells was developed and optimized based on an enzymatic digestion method. Briefly, the conjunctiva of eight-week-old to 12-week-old SWR/J mice were cut into fragments and incubated in RPMI1640 medium supplemented with 10% fetal bovine serum, 1.5 mg/ml collagenase (Nitta Gelatin, Osaka, Japan), 0.5 mg/ml hyaluronidase (Sigma), and 0.5 mg/ml DNase I (Sigma) for 2 h at 37 °C. The dispersed cells were filtered through a 40 μm cell strainer, layered on an isotonic Percoll density medium (density=1.041, Amersham Pharmacia Biotech, Piscataway, NJ), and centrifuged at 800x g for 20 min [11]. The pellet was cultured in RPMI1640 with murine recombinant IL-3 (10 ng/ml; Peprotech, Rocky Hill, NJ), recombinant SCF (10 ng/ml; Peprotech), and 5% serum for two days. Colony-forming, non-adherent mast cells were harvested by gentle shaking and decanting of the flasks. The cells were then layered onto a Percoll density medium (density=1.041) and centrifuged at 800x g for 20 min.

To precisely evaluate the roles of mast cells, a further purification protocol was developed for the functional analyses. Based on preliminary FACS analyses, contaminated populations included dendritic cells, macrophages, and plasma cells, which were characterized by the expression of CD4, CD8a, CD11b, B220, and PDCA-1. These markers were selected and confirmed for appropriateness for depletion markers based on the co-expression of mature mast cell markers such as CCR3 and CXCR3 (data not shown). To deplete these cells, we used a magnet–based negative depletion method to obtain maximum recovery. Briefly, cells were blocked by anti-CD32 (Clone 93; eBiosciences, San Diego, CA) and labeled with rat anti-CD4, rat anti-CD8a, rat anti-CD11b, rat anti-B220 (all from eBiosciences), and rat anti-PDCA-1 (Miltenyi Biotec Inc., Auburn, CA). The labeled fractions were depleted using anti-rat immunoglobulin κ chain antibody-conjugated microbeads (IMag; BD Biosciences).

For FACS analyses, cultured mast cells were blocked by anti-CD32 and stained for immunofluorescence analysis. The analysis was performed with the FACSCalibur flow cytometer (Becton Dickinson, Franklin Lakes, NJ). Phycoerythrin (PE)-conjugated anti-CXCR3 antibody (R&D Systems, Minneapolis, MN), biotin-conjugated anti-c-Kit antibody (2B8; eBiosciences), PE-conjugated anti-c-Kit antibody (eBiosciences), FITC-conjugated-anti-FcεRI α subunit (MAR-1, eBiosciences), and Peridinin-chlorophyll-protein (PerCP)-conjugated streptavidin (BD Biosciences) were used for staining the mast cells. FITC-conjugated IgG (eBiosciences), PE-conjugated IgG (eBiosciences), PerCP-conjugated IgG (BD Biosciences), and biotin-conjugated IgG (eBiosciences) were used for isotype control staining.

### Adoptive transfer of isolated conjunctival mast cells

Purified mast cells (4×10^5^ cells/eye) from wild type mice were injected subconjunctivally into allergen-sensitized, mast cell-deficient *Kit^w^/Kit^w-v^* mice. Immediate hypersensitivity was induced by allergen exposure two weeks after the transfer. Connective tissue type mast cells were identified in the recipient mice and control wild type mice by measuring the conjunctival transcripts of mMCP-5, mMCP-6, and FcεRI α subunit by quantitative reverse transcription polymerase chain reaction (RT–PCR) and Giemsa staining.

### Statistical analyses

Data are presented as the means±standard error of the means (SEMs). Statistical analyses were performed by ANOVA.

## Results

### Mast cells required for early phase response of allergic conjunctivitis

We first examined whether the tissues on the ocular surface of *Kit^w^/Kit^w-v^* mice had any mature mast cells that could be identified by the presence of metachromatic granules. An earlier investigation showed that mast cells were not present in different mucosal tissues of these mice [6], and in confirmation, we found that mast cells were absent in the conjunctiva, eyelids, and choroid of naïve *Kit^w^/Kit^w-v^* mice.

**Figure 1:**
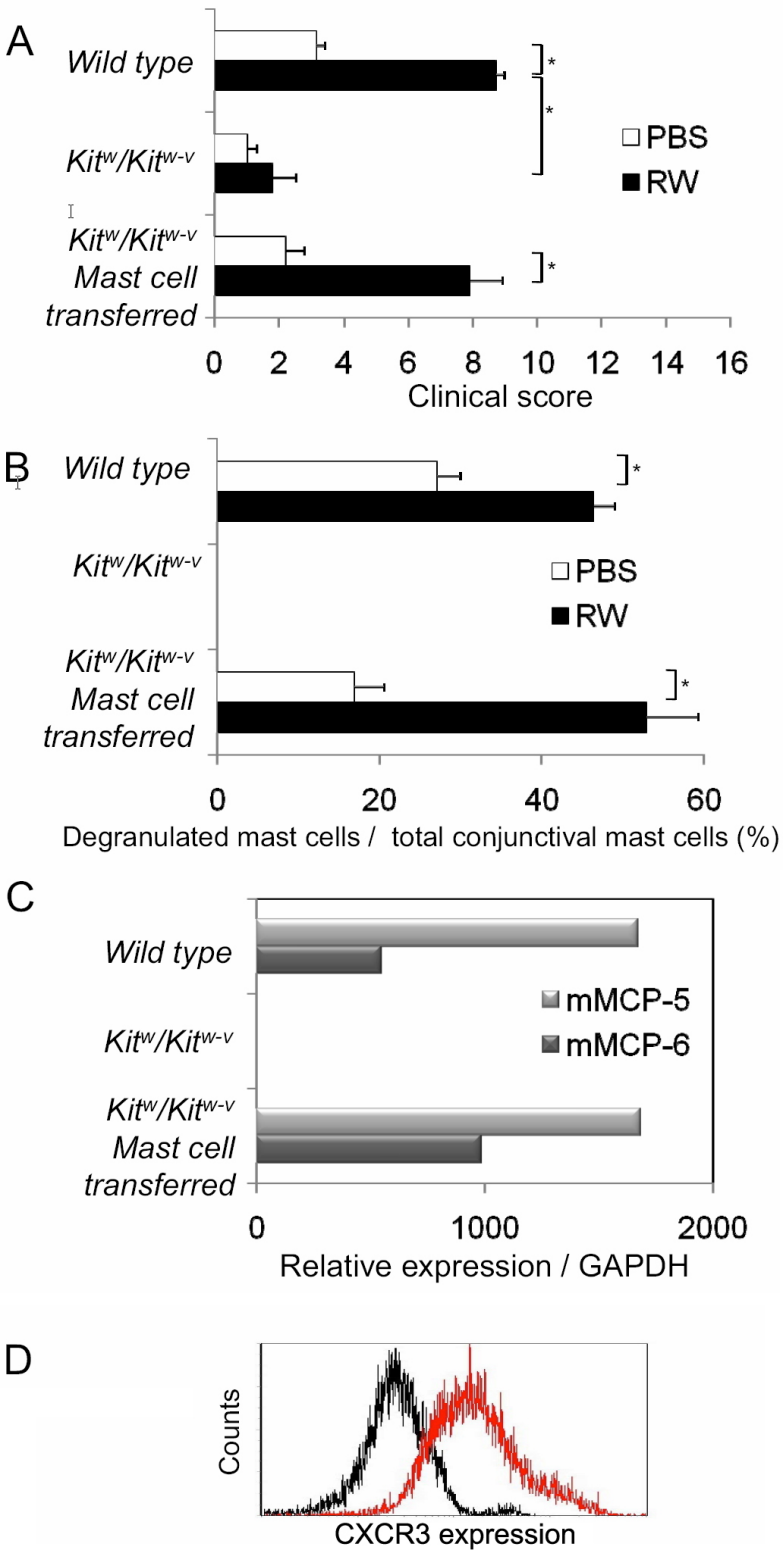
Effect of adoptive transfer of mast cells isolated from wild type mice to mast cell-deficient mice on the clinical responses in the acute phase. **A**: Recovery of defective acute phase clinical responses in *Kit^w^/Kit^w-v^* mice after adoptive subconjunctival transfer of conjunctival mast cells from wild type cells is shown. The clinical scores for both sensitized and mock-sensitized *Kit^w^/Kit^w-v^* mice were significantly lower following allergen challenge than that of wild type mice. **B**: Allergen-induced-mast cell degranulation in *Kit^w^/Kit^w-v^* mice after adoptive transfer of conjunctival mast cells from wild type mice is demonstrated in the chart. Mast cell degranulation in the conjunctiva, undetectable in *Kit^w^/Kit^w-v^* mice, was observed in both sensitized and mock-sensitized adoptive transfer mice. **C**: Expression of mast cell-restricted proteases, mMCP-5 and mMCP-6, in the conjunctiva of *Kit^w^/Kit^w-v^* mice after adoptive transfer is shown.  Mice injected with the conjunctival mast cells had levels of mMCP-5 and mMCP-6 that were comparable to those of wild type mice. **D**: Expression of CXCR3 as a maturation marker on conjunctiva-derived FcεRI^+^ c-Kit^+^ mast cells by FACS analysis is demonstrated.  Red indicates the CXCR3 stained conjunctiva-derived mast cells. Black shows the isotype control. RW: sensitized with ragweed pollen; PBS: mock sensitized; n=10 mice/group.

To evaluate the contribution of mast cells to the ocular symptoms during the early phase allergic response, we challenged sensitized mice with the allergen following the single exposure protocol. Mice were assessed for clinical signs including conjunctival edema, conjunctival redness, tearing, and lid edema. The degree of inflammation during the early phase is summarized as clinical scores in Figure 1A. The clinical scores for both sensitized and mock-sensitized *Kit^w^/Kit^w-v^* mice were significantly lower following the allergen challenge than that of wild type mice (sensitized wild type: 8.7±0.3, sensitized *Kit^w^/Kit^w-v^*: 1.8±0.7, p<0.05). The clinical scores for *Kit^w^/Kit^w-v^* mice appeared not specific for the allergen because no significant difference was observed between the sensitized and mock-sensitized mice. This is consistent with the concept that conjunctival mast cells mediate the clinical signs after the allergen challenge.

To examine the contribution of mast cells to the clinical signs in allergic inflammation in more detail, we restored conjunctival mast cells to the mast cell-deficient *Kit^w^/Kit^w-v^* mice. Conjunctival mast cells, isolated by lineage marker depletion after density-gradient separation, were tested for allergen-induced degranulation and mast cell-restricted proteases. The conjunctiva-derived mast cells expressed high levels of the connective tissue type mast cell proteases, mMCP-5, mMCP-6, and mMCP-7. When these cells were sensitized with anti-DNP-specific IgE and exposed to DNP-albumin, they appropriately degranulated in an allergen-specific manner (data not shown).

Peripheral tissues, including the conjunctiva, are continuously replenished by the migration of bone marrow-derived mast cells. To exclude any effect of newly immigrated cells from the bone marrow, the conjunctiva of mast cell-deficient mice were reconstituted with the conjunctiva-derived mast cells from the wild type mice.

The conjunctival mast cells that were expressing CXCR3 as a maturation marker of conjunctival mast cells (Figure 1D) [5] and isolated from naive wild type mice were adoptively transferred into the allergen-sensitized *Kit^w^/Kit^w-v^* mice by subconjunctival injection. Conjunctival expression of the mast cell restricted transcripts, mMCP-5 and mMCP-6, was used to assess the success of the transfer. As expected, mMCP-5 and mMCP-6 were not expressed at appreciable levels in the conjunctiva of *Kit^w^/Kit^w-v^* mice (Figure 1C). In contrast, mice injected with the conjunctival mast cells had levels of mMCP-5 and mMCP-6 that were comparable to those of wild type mice (Figure 1C). This confirmed that the transferred mast cells maintained the properties of the connective tissue type as conjunctival mast cells.

To examine their homing ability, real time PCR was conducted to examine levels of mast cell-specific proteases in the draining lymph nodes of the adoptive transfer mice. Neither mMCP-5 nor mMCP-6 was detected in the adoptive transfer mice (below detection limits). This indicated that the transferred mast cells remained in the conjunctiva and did not migrate elsewhere.

When the adoptive, transferred *Kit^w^/Kit^w-v^* mice were challenged with an allergen, clinical signs of allergic inflammation were restored (Figure 1A). Mast cell degranulation in the conjunctiva, undetectable in *Kit^w^/Kit^w-v^* mice, was observed in both sensitized and mock-sensitized adoptive transfer mice. The sensitized mice had significantly more allergen-specific mast cell degranulation than the mock-sensitized mice (53% versus 17%, p<0.05; Figure 1B). The restored response was indistinguishable from that of the wild type mouse response, supporting the validity of our adoptive transfer model using *Kit^w^/Kit^w-v^* mice with conjunctival mast cells.

We have shown that the degree of mast cell degranulation is linearly correlated with the clinical scores [9]. Thus, conjunctival mast cells are necessary and sufficient for activation of the acute phase clinical responses.

**Figure 2:**
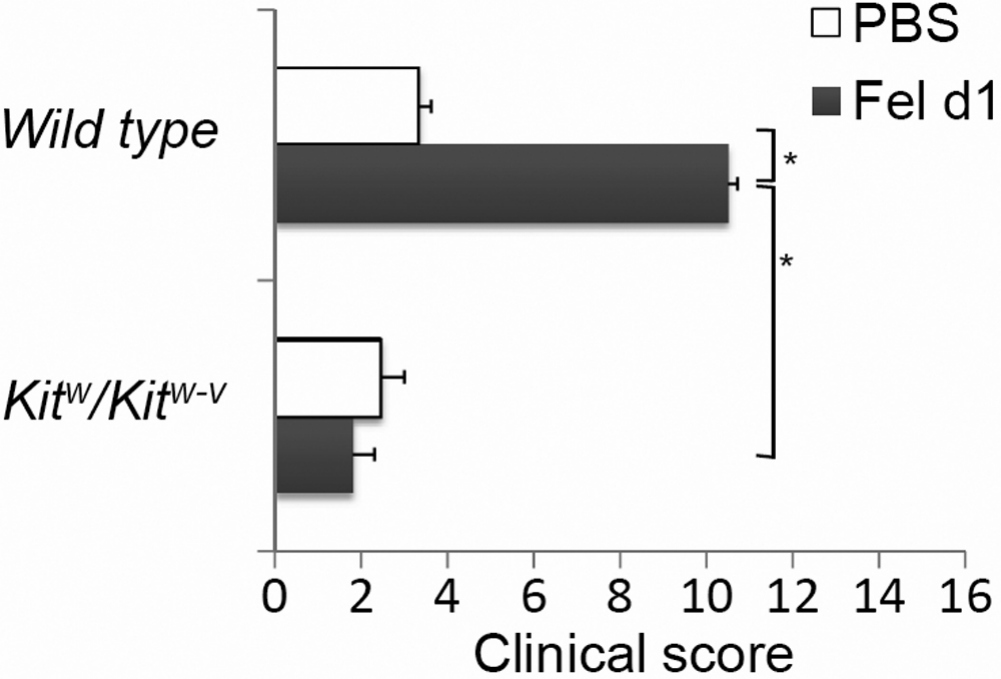
Abolishment of early phase clinical symptoms in mast cell-deficient mice. The clinical scores were also significantly lower in the sensitized mast cell-deficient mice than in sensitized wild type mice following multiple allergen exposures. An asterisk indicates that p<0.005. Fel d 1: sensitized with Fel d 1; PBS: mock-sensitized; n=10 mice/group.

In clinical settings, allergic symptoms are provoked after repeated exposure to an allergen. We have shown that repeated exposure to an allergen aggravated the clinical signs and altered mast cell restricted proteases [12]. Therefore, we tested whether repeated allergic exposure could stimulate mast cell recruitment or maturation to correct the conjunctival mast cell deficiency in *Kit^w^/Kit^w-v^* mice. Our results showed that even after repeated allergen exposures, conjunctival mast cells were not detected in sensitized *Kit^w^/Kit^w-v^* mice. The clinical scores were also significantly lower in the sensitized mast cell-deficient mice than in sensitized wild type mice following multiple allergen exposures, (1.8 versus 10.5, p<0.05; Figure 2). The clinical signs were completely suppressed, and the tear/discharge score was depressed by 78%.

To determine whether mast cell deficiency affected antigen-specific B cell and T cell responses during allergic conjunctivitis, levels of allergen-specific IgE and IgG_1_ were measured after the final allergen challenge. The titers of IgE and IgG reflect the function of both B cells and T cells because an increased production of IgE and IgG_1_ requires numerous B cell and T cell processes including antigen presentation, antigen-specific T cell expansion, class switch recombination of B cells, and specific antibody production.

**Figure 3:**
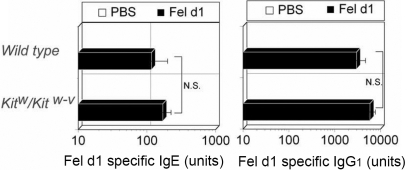
Induction of Fel d1-specific IgE and IgG1 in *Kit^w^/Kit^w-v^* mice. *Kit^w^/Kit^w-v^* mice are not impaired for Fel d 1-specific IgE or IgG_1_. Fel d 1: sensitized with Fel d 1; PBS: mock-sensitized; n=10 mice/group.

Allergen-specific IgE and IgG_1_ were appropriately induced following the allergen challenge in sensitized wild type mice but not in mock-sensitized wild type mice (Figure 3). Levels of allergen-specific IgE and IgG_1_ were not significantly different between *Kit^w^/Kit^w-v^* mice and wild type mice, indicating that the inductive arm of *Kit^w^/Kit^w-v^* mice was still functioning normally during the sensitization phase.

### Requirement of mast cells for late phase response of allergic conjunctivitis

**Figure 4:**
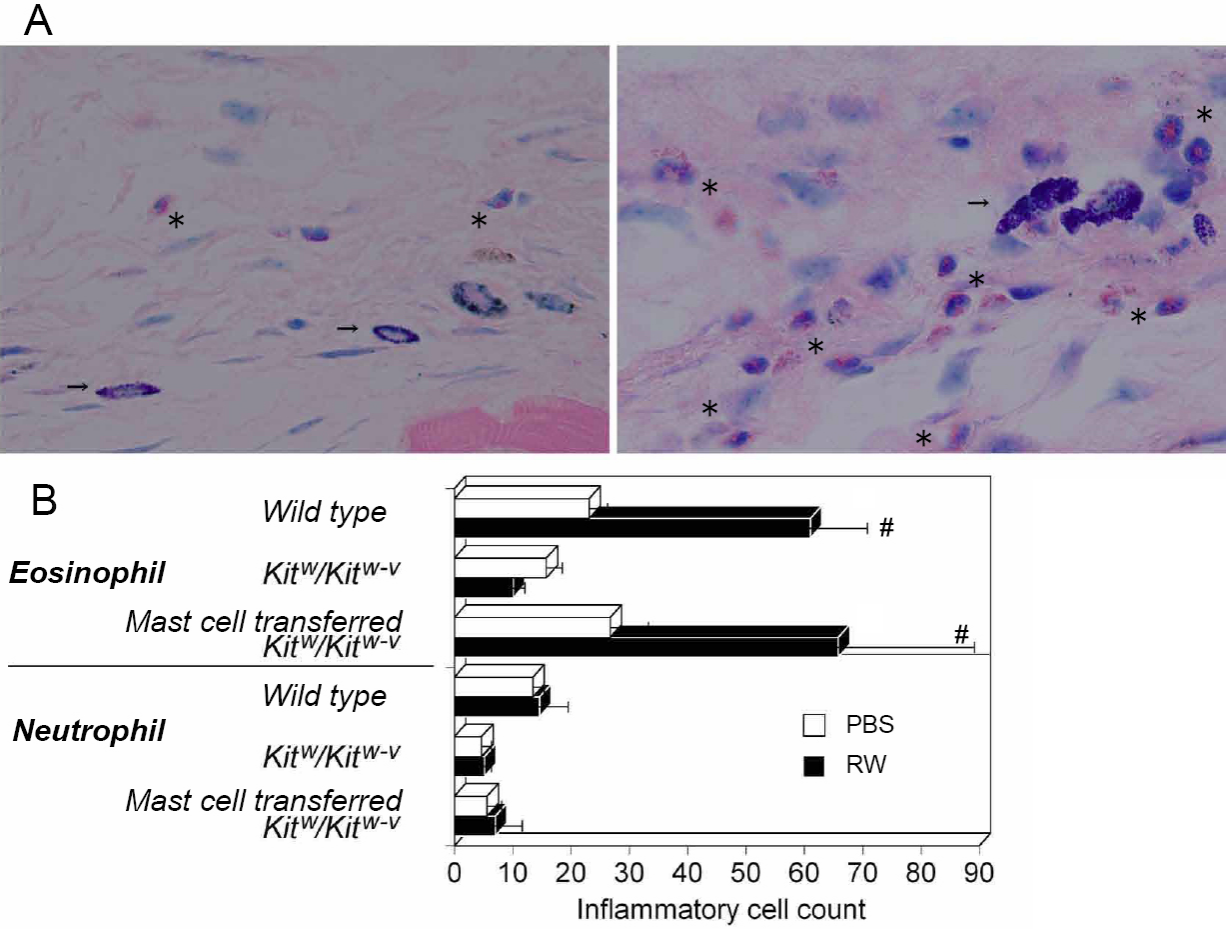
Impairment of late phase inflammatory responses in *Kit^w^/Kit^w-v^* mice and subsequent response recovery in *Kit^w^/Kit^w-v^* mouse recipients of conjunctival mast cells. **A**: *Kit^w^/Kit^w-v^* mice, subconjunctivally injected with wild type conjunctival mast cells, were challenged with an allergen, and the conjunctiva were processed for Giemsa staining after 24 h. The left image shows conjunctiva of mock-sensitized *Kit^w^/Kit^w-v^* adoptive transfer mice. The right image shows conjunctiva of allergen-sensitized *Kit^w^/Kit^w-v^* adoptive transfer mice. Each asterisk in the images denotes the presence of eosinophils, and an arrow indicates transferred mast cells. **B**: Recovery of defective eosinophil recruitment in *Kit^w^/Kit^w-v^* mice by subconjunctival adoptive transfer of conjunctival mast cells from wild type mice is shown in the chart. The sharp (hash mark) means that p<0.05. RW: sensitized with ragweed pollen; PBS: mock sensitized; n=10 mice/group.

We evaluated the dependency of the late phase inflammatory response on mast cells by examining recruitment of inflammatory cells 24 h after the allergen challenge. To exclude the possible effects of preconditioning by repeated exposures, we first used the single exposure protocol for sensitization. Allergen-specific, late phase responses in wild type mice were characterized mainly by conjunctival infiltration of eosinophils. Foci of inflammatory cells were accompanied or surrounded by mast cells, implying some role for the mast cells in this process (Figure 4A). No increase in conjunctival infiltration of eosinophils and neutrophils was observed in sensitized *Kit^w^/Kit^w-v^* mice 24 h after the allergen challenge (Figure 4B). However, when conjunctival mast cells were transferred to sensitized *Kit^w^/Kit^w-v^* mice, the allergen-specific eosinophil recruitment in the transferred mice was completely restored (Figure 4B). These observations clearly demonstrated that the late phase eosinophil infiltration is dependent on mast cell activity. Interestingly, we did not observe an increase in neutrophil recruitment in any of the sensitized animals compared to their mock-sensitized counterparts (Figure 4B).

**Figure 5:**
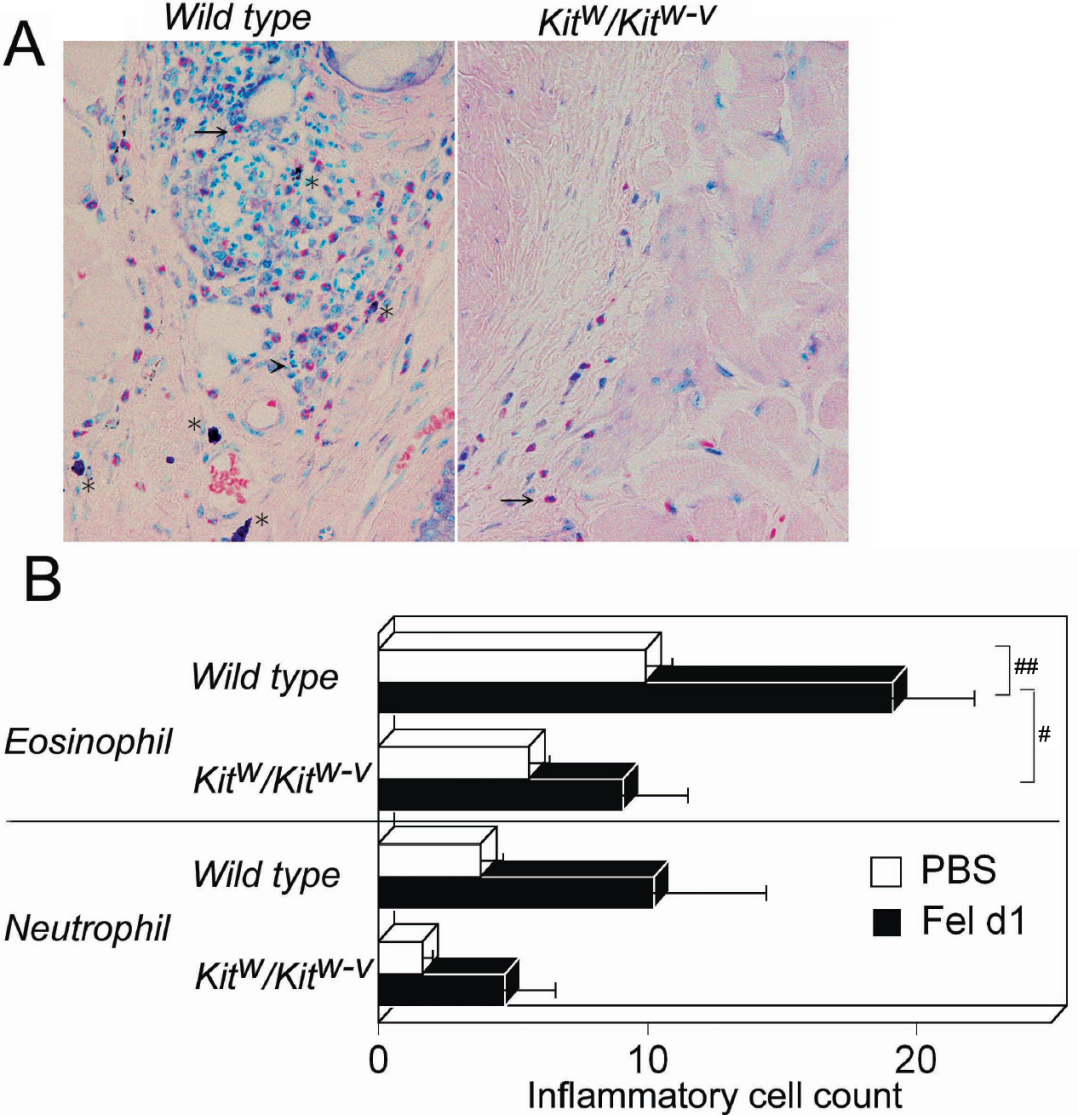
Impairment of late phase inflammatory responses in *Kit^w^/Kit^w-v^* mice. **A**: *Kit^w^/Kit^w-v^* mice were challenged with an allergen, and the conjunctiva were processed for Giemsa staining after 24 h. The asterisk in the image denotes mast cells, and an arrow denotes the presence of eosinophils. 400X magnification. **B**: Recruitment of eosinophils and neutrophils in the conjunctiva of *Kit^w^/Kit^w-v^* mice is show in the chart. The sharp (hash mark) denotes that p<0.005 and the double sharp denotes that p<0.05. Fel d1: sensitized with Fel d1; PBS: mock sensitized; n=10 mice/group.

We next sensitized mice using the repeated allergen exposure model [9] and then evaluated mast cell contributions to the local activation or preconditioning of bystander cells or mast cells. In wild type mice, an allergen challenge significantly increased the number of conjunctival eosinophils in the sensitized animals but not in the mock-sensitized animals (Figure 5). However, the number of conjunctival eosinophils was not significantly increased in sensitized *Kit^w^/Kit^w-v^* mice (Figure 5B). The conjunctival eosinophil count following allergen exposure was significantly lower in *Kit^w^/Kit^w-v^* mice than in wild type mice (19.1 versus 9.0, p<0.005), although there was no significant difference in the number of eosinophils between the mock-sensitized wild type and *Kit^w^/Kit^w-v^* mice. All changes in the neutrophil count following the allergen challenge were not significant (Figure 5B).

Taken together, the data from the adoptive transfer model have shown that conjunctival mast cells are essential for eosinophil recruitment in the early phase and late phase allergic responses in the eye but not essential for neutrophil recruitment.

## Discussion

Our results demonstrated important aspects of mast cell involvement in ocular allergy (i.e., the activation of locally residing conjunctival mast cells) after an allergen exposure induced the recruitment of eosinophils. This is in marked contrast to the conclusions of Ueta et al. [4] who reported that mast cells were not required for eosinophil recruitment in allergic conjunctivitis using antigen-sensitized, mast cell-deficient mice. However, their conclusion considered only one aspect of allergic conjunctivitis. It is known that allergic conjunctivitis is triggered by the mast cell degranulation-mediated cascade, and this needs to be clearly shown in the model being evaluated for mast cell dependence. However, eosinophilia is a good indicator of allergic conjunctivitis. Indeed, the mast cell-mediated activation, which was not presented in their report, primes Th2-type T cells-dependent eosiophil recruitment. For example, the transfer of Th2-type T cells can induce massive eosinophilic inflammation. Also, in the corneal transplantation model, inflammatory responses can be eosinophilic in certain donor and recipient combinations [13]. Thus, one can create experimental settings where the involvement of mast cells is least required. Evaluation of eosinophilic inflammation in allergic conjunctivitis needs to be evaluated by the activation of mast cells.

We do not argue that eosinophilia can occur with a minimal activation of mast cells. This situation can be created by repetitive and strong immunizations. Ueta et al. [4] used an intradermal injection of ragweed allergen followed by multiple boosting by two intraperitoneal injections and four repetitive allergen exposures. This would usually result in a vigorous T cell-mediated response and clonal expansion that should culminate in a massive eosinophil recruitment after an antigen challenge [3,14,15]. In addition, they claimed that the attenuated protocol (one intradermal injection followed by one intraperitoneal injection) still provoked allergen-induced eosinophil recruitment in mast cell-deficient mice. However, this model also appeared to be independent of IgE-mediated mast cell degranulation because no allergen-specific IgE was detected in the sera of their experimental mice.

The contribution of mast cells to the eosinophilic responses can be explained by the properties of the cytokines and chemokines released upon activation. For example, conjunctival mast cells activated by FcεRI produce IL-2, IL-3, IL-4, IL-5, IL-6, IL-10, IL-12, GM-CSF, MIP-1α, and TNF-α (unpublished observations). IL-3 and IL-5 are particularly likely to contribute to eosinophilic inflammation as they play critical roles in eosinophil development, survival, and recruitment [16]. Other mast cell-derived mediators that might contribute to eosinophil activity in ocular hypersensitivity include the leukotrienes, B4 and D4, and tryptases [17,18]. These observations are consistent with the idea that T cell priming is required for mast cell-related eosinophilia and support our findings that mast cells mediated the eosinophil recruitment in the late phase inflammation.

It has been suggested that the role of conjunctival mast cells was to induce neutrophilic inflammation. For example, when mast cell degranulation is induced in the conjunctiva by compound 48/80, the resulting conjunctivitis is characterized by infiltration of neutrophils, macrophages, and CD4^+^ T lymphocytes but surprisingly few eosinophils [19]. This is in marked contrast to our observations, which showed that neutrophilic inflammation does not require conjunctival mast cells. This might be due to the relative low levels of neutrophilic cytokines derived from mature mast cells. The mononuclear cells and macrophages are the major sources of neutrophilic cytokines such as TNF-α and MIP-1α. We suggest that the neutrophilic inflammation induced by compound 48/80 may be explained by such mediators derived from mononuclear cells and macrophages, which are indirectly stimulated by mast cell-derived chemokines or cytokines.

In conclusion, our new mast cell reconstituted model is a better method to investigate what role conjunctiva-residing mast cells play in experimental allergic conjunctivitis. This allowed us to evaluate the direct contribution of mast cells to eosinophilic inflammation. Our data support the idea that regulation of mast cell activation and maturation in addition to current therapy to suppress mast cell degranulation might be promising therapeutic strategies. Direct targeting of c-Kit, mast cell-derived chemokines, or chemokine receptors might also yield good therapeutic results [9,20-22].
